# Defining a Novel Role for the Coxsackievirus and Adenovirus Receptor in Human Adenovirus Serotype 5 Transduction *In Vitro* in the Presence of Mouse Serum

**DOI:** 10.1128/JVI.02487-16

**Published:** 2017-05-26

**Authors:** Estrella Lopez-Gordo, Andor Doszpoly, Margaret R. Duffy, Lynda Coughlan, Angela C. Bradshaw, Katie M. White, Laura Denby, Stuart A. Nicklin, Andrew H. Baker

**Affiliations:** a Institute of Cardiovascular and Medical Sciences, BHF Glasgow Cardiovascular Research Centre, University of Glasgow, Glasgow, United Kingdom; b Centre for Cardiovascular Science, Queen's Medical Research Institute, University of Edinburgh, Edinburgh, United Kingdom; c Department of Oncology, University of Oxford, Oxford, United Kingdom; d The Jenner Institute, Nuffield Department of Medicine, University of Oxford, Oxford, United Kingdom; The Biodesign Institute, Arizona State University

**Keywords:** adenovirus, CAR, receptor, serum, tropism

## Abstract

Human adenoviral serotype 5 (HAdV-5) vectors have predominantly hepatic tropism when delivered intravascularly, resulting in immune activation and toxicity. Coagulation factor X (FX) binding to HAdV-5 mediates liver transduction and provides protection from virion neutralization in mice. FX is dispensable for liver transduction in mice lacking IgM antibodies or complement, suggesting that alternative transduction pathways exist. To identify novel factor(s) mediating HAdV-5 FX-independent entry, we investigated HAdV-5 transduction *in vitro* in the presence of serum from immunocompetent C57BL/6 or immunocompromised mice lacking IgM antibodies (Rag 2^−/−^ and NOD-scid-gamma [NSG]). Sera from all three mouse strains enhanced HAdV-5 transduction of A549 cells. While inhibition of HAdV-5–FX interaction with FX-binding protein (X-bp) inhibited transduction in the presence of C57BL/6 serum, it had negligible effect on the enhanced transduction observed in the presence of Rag 2^−/−^ or NSG serum. Rag 2^−/−^ serum also enhanced transduction of the FX binding-deficient HAdV-5HVR5*HVR7*E451Q (AdT*). Interestingly, Rag 2^−/−^ serum enhanced HAdV-5 transduction in a FX-independent manner in CHO-CAR and SKOV3-CAR cells (CHO or SKOV3 cells transfected to stably express human coxsackievirus and adenovirus receptor [CAR]). Additionally, blockade of CAR with soluble HAdV-5 fiber knob inhibited mouse serum-enhanced transduction in A549 cells, suggesting a potential role for CAR. Transduction of HAdV-5 KO1 and HAdV-5/F35 (CAR binding deficient) in the presence of Rag 2^−/−^ serum was equivalent to that of HAdV-5, indicating that direct interaction between HAdV-5 and CAR is not required. These data suggest that FX may protect HAdV-5 from neutralization but has minimal contribution to HAdV-5 transduction in the presence of immunocompromised mouse serum. Alternatively, transduction occurs via an unidentified mouse serum protein capable of bridging HAdV-5 to CAR.

**IMPORTANCE** The intravascular administration of HAdV-5 vectors can result in acute liver toxicity, transaminitis, thrombocytopenia, and injury to the vascular endothelium, illustrating challenges yet to overcome for HAdV-5-mediated systemic gene therapy. The finding that CAR and potentially an unidentified factor present in mouse serum might be important mediators of HAdV-5 transduction highlights that a better understanding of the complex biology defining the interplay between adenovirus immune recognition and cellular uptake mechanisms is still required. These findings are important to inform future optimization and development of HAdV-5-based adenoviral vectors for gene therapy.

## INTRODUCTION

Vectors based on human adenoviral serotype 5 (HAdV-5) are promising for gene therapy and vaccination applications. However, although HAdV-5-based vectors are widely used in clinical trials (http://www.wiley.com//legacy/wileychi/genmed/clinical/), their use for gene therapy following intravascular delivery is limited by high hepatic tropism, which leads to activation of antiviral immune responses and toxic side effects (1–4). While the classical *in vitro* pathway for HAdV-5 transduction is primarily via the capsid fiber protein binding to the coxsackievirus and adenovirus receptor (CAR) and subsequent internalization via the capsid penton base engaging α_v_β_3,5_ integrins (5–8), the *in vivo* entry pathway is still being elucidated in detail.

Previous studies have reported host cell receptors and factors that dictate HAdV-5 tropism. Coagulation factor X (FX) was identified as the key factor mediating HAdV-5 liver transduction (9). FX binds to the capsid hexon proteins in 1:1 stoichiometry at nanomolar affinity and bridges HAdV-5 to heparan sulfate proteoglycan (HSPG) on hepatocytes leading to hepatic transduction (9–11). FX binds to the HAdV-5 hexon hypervariable regions (HVRs) through its γ-carboxyl glutamic acid (GLA) domain while also binding to the *N*- and *O*-linked sulfate groups of HSPGs via the serine protease (SP) domain (10–15). Genetic engineering of HAdV-5 to ablate the key residues in the hexon HVRs that interact with FX greatly diminished liver transduction (12, 16), highlighting the key role this pathway plays in mediating hepatic gene delivery. More recently, an alternative role for the HAdV-5–FX interaction was defined, which involves FX capsid coating or “shielding,” resulting in protection of the virus from neutralization or “immune attack” (17–19). Adenoviral neutralization was shown to be mediated by natural immunoglobulin M (IgM) antibodies and the complement system present in mouse serum, which block binding of virions to host cells and thus prevent transduction (17). In particular, FX was shown to prevent binding of human IgM antibodies to virions (19) and activation of mouse C3 (complement component 3) convertase and subsequent covalent modification of virions with C3 (17). It was also demonstrated that hepatic tropism of HAdV-5 did not require HAdV-5–FX interaction in immunocompromised mice lacking IgM antibodies, complement protein C1q, or complement component 4 (C4) (17). Furthermore, a recent study reported that while HAdV-5 uses the FX- and HSPG-mediated transduction pathway in primary hepatocytes *in vitro*, *in vivo* liver transduction was not significantly reduced in mice lacking *Ext1*, an enzyme required for heparan sulfate (HS) biosynthesis (20). Thus, these recent reports suggest the involvement of other unidentified alternative transduction mechanisms for HAdV-5 when the FX-mediated pathway is unavailable. These alternative transduction pathways may be mediated directly via HAdV-5 engaging with cellular receptors or by bridging to intermediate molecules.

Many studies have attempted to identify specific cell surface receptors mediating HAdV-5 tropism. Direct interactions between adenoviral vectors and heparan sulfate glycosaminoglycan (HS-GAG) have demonstrated that HS-GAG can facilitate HAdV-5 binding and infectivity of A549 and HeLa cells *in vitro* (21, 22). However, genetic mutations to ablate adenoviral binding to HSPG (23–25) or the use of mouse models that lack heparan sulfate (20) failed to achieve liver detargeting. Although other studies have shown that mutation of the fiber shaft to ablate a putative HSPG-interacting motif could reduce liver transduction (26, 27) and cell transduction *in vitro* (25), it is now widely believed that these effects are possibly due to alterations in the fiber structure, conferring rigidity and thus hampering simultaneous binding to CAR and α_v_β_3,5_ integrins and affecting trafficking of virions, rather than modulating direct binding to HSPG (25, 28). Moreover, ablating the ability of HAdV-5 to interact with CAR or α_v_β_3,5_ integrins has for the most part failed to achieve efficient liver detargeting (20, 23, 26, 27, 29–34). However, in these studies, genetic mutations in individual capsid proteins (fiber and penton base) were assessed, thus not addressing the possibility that HAdV-5 may use as yet unidentified circulating blood factors to interact with cell surface receptors via a bridging mechanism.

Indeed, HAdV-5 has been previously reported to interact with several circulating blood proteins such as C4-binding protein (C4BP) (35), coagulation factor VII (FVII) (9), coagulation factor IX (FIX) (9, 10, 35), and protein C (PC) (9, 10). Despite FVII being able to bind to HAdV-5 and containing a heparin-binding exosite (36, 37), it may be unable to interact with HSPG when forming a complex with HAdV-5 due to the formation of dimers between the FVII SP domains (37). FIX also binds to HAdV-5 (10), and like FVII, it has a heparin-binding exosite (38, 39). However, no evidence of FIX dimer formation has yet been described, suggesting FIX might potentially bridge HAdV-5 to HS for cell transduction. Indeed, FIX has been reported to enhance binding to and infection of epithelial cells with HAdV-18 (40) and with HAdV-31 through HS-GAG (41). Furthermore, FIX enhanced HAdV-5-mediated transduction of mouse hepatocytes and Kupffer cells *in vitro* and *in vivo* and human hepatocytes *in vitro* (35). C4BP has also been reported to confer CAR-independent adenoviral transduction of primary human hepatocytes *in vitro* (35), while PC was shown to mediate HepG2 hepatocyte transduction (9).

To investigate possible HAdV-5 transduction pathways involving interactions with bridging molecules that may be relevant *in vivo*, in the present study, we evaluated HAdV-5 transduction *in vitro* in the presence or absence of mouse serum from immunocompetent or immunocompromised strains. Our findings suggest that HAdV-5 vectors exposed to mouse serum are able to use different mechanisms of cell entry. In addition to the well-known FX-mediated pathway of HAdV-5 cell entry, we report use of a FX-independent and CAR-mediated mechanism for HAdV-5 transduction *in vitro* in the presence of mouse serum, which is independent of fiber knob interaction with CAR.

## RESULTS

### Serum from immunocompromised mice enhances HAdV-5 transduction *in vitro* via a FX-independent mechanism.

**FIG 1:**
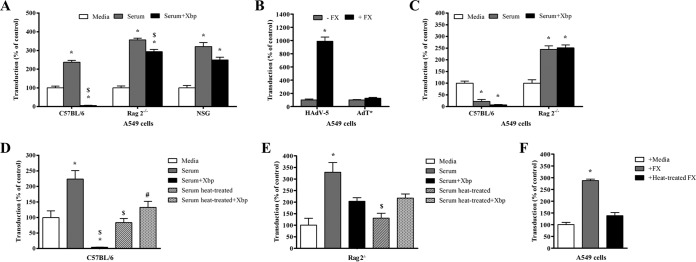
Effect of mouse serum or coagulation factor X (FX) on adenoviral transduction. (A and C to F) HAdV-5 or AdT* (FX binding-deficient) (2 × 10^10^ vp/ml) was incubated for 30 min at 37°C with serum-free (SF) media, 90% C57BL/6, Rag 2^−/−^, or NSG serum in the presence or absence of X-bp (40 μg/ml) (A and C to E) or with human FX (hFX) (10 μg/ml) in SF media (F). When indicated, hFX or mouse serum was preincubated at 56°C for 30 min before the addition of X-bp (D to F). Adenovirus suspensions were added to A549 cells (MOI of 1,000 vp/cell) and incubated at 37°C for 2 h. The medium was then replaced with media containing 2% FCS, and the cells were incubated for another 20 h. (B) A549 cells were incubated with HAdV-5 or AdT* (MOI, 1,000 vp/cell) at 37°C for 3 h in the presence (+) or absence (−) of hFX (10 μg/ml), the medium was replaced with 10% FCS-containing media, and the cells were incubated for further 48 h. β-Galactosidase expression levels were quantified as relative light units (RLU) and normalized to the total milligrams of protein (A to F). There were four technical replicates per condition (B), pooled data from seven (C57BL/6), six (Rag 2^−/−^), or three (NSG) mice (A and C), or three (D to F) independent experiments with four replicates per condition. Values are shown as a percentage of the SF-medium-alone condition and expressed as the mean of values (B) or of the normalized values per experiment (A and C to F) plus standard error of the mean (SEM) (error bar). Repeated-measures ANOVA and *posthoc* Tukey's range test (A and C to F) or unpaired Student's *t* test (B) were applied. Values that are significantly different are indicated as follows: *, *P* < 0.05 versus matched controls; $, *P* < 0.05 versus serum; #, *P* < 0.05 versus serum plus X-bp.

To study the effect of mouse serum on adenoviral transduction of cells *in vitro*, transduction assays were performed in A549 cells using HAdV-5 β-galactosidase, with the addition of serum from immunocompetent or immunocompromised mice. As expected, adenoviral transduction occurred at low levels in the presence of serum-free media alone (Fig. 1A). Immunocompetent C57BL/6 serum significantly enhanced HAdV-5 transduction by approximately 2.4-fold (Fig. 1A), highlighting the presence of a factor(s) in serum that enhances HAdV-5 transduction. Preincubation of C57BL/6 serum with FX-binding protein (X-bp), a molecule that suppresses HAdV-5–FX interactions by specifically binding to the FX GLA domain (42), substantially inhibited transduction (Fig. 1A). These data are in agreement with previous reports showing that incubation of HAdV-5 with C57BL/6 serum in the absence of HAdV-5–FX binding results in loss of transduction and is consistent with the hypothesis of natural IgM antibodies and complement-mediated adenovirus neutralization inhibiting HAdV-5 binding to host cells (17, 18).

Next, to assess whether FX is involved in HAdV-5 transduction of cells *in vitro* in the absence of adenovirus neutralization, transduction assays were performed with serum from immunocompromised Rag 2^−/−^ mice. These mice are unable to differentiate B and T lymphocytes to a mature state, thus lacking IgM antibodies (43, 44), one of the mediators of HAdV-5 neutralization *in vitro* in the absence of HAdV-5–FX binding (17). Similar to observations with C57BL/6 serum, Rag 2^−/−^ serum significantly enhanced HAdV-5 transduction by approximately 3.5-fold (Fig. 1A). In contrast to C57BL/6 serum, Rag 2^−/−^ serum preincubated with X-bp did not inhibit HAdV-5 transduction (Fig. 1A), showing that Rag 2^−/−^ serum was unable to neutralize HAdV-5. Preincubation of Rag 2^−/−^ serum with X-bp resulted in a small decrease in HAdV-5 transduction compared to transduction in the absence of X-bp (Fig. 1A), indicating that FX may only partially contribute to the enhanced cell transduction observed with Rag 2^−/−^ serum. Importantly, this reduction was substantially less than that observed in the presence of C57BL/6 mouse serum and X-bp (Fig. 1A). To dismiss possible individual mouse strain-specific effects, these results were confirmed using serum from NOD-scid-gamma (NSG) mice, another immunocompromised mouse strain (Fig. 1A). These mice are not able to produce mature B or T lymphocytes, are defective in dendritic cell and macrophage cytokine production and hemolytic complement protein 5 (C5), and have extremely low natural killer (NK) cell cytotoxic activity (45). NSG mice were also chosen due to their lack of IgM antibody production (46). The transduction profile for HAdV-5 in the presence of NSG serum matched that of Rag 2^−/−^ serum.

To further confirm HAdV-5 transduction in the presence of immunocompromised mouse serum was via a FX-independent mechanism, the FX binding-deficient AdT* (12) was used in comparison to HAdV-5. Lack of sensitivity to FX was confirmed for AdT* by assessing cell transduction in the presence or absence of FX in A549 or SKOV3 cells, which express high or low levels of CAR on the plasma membrane, respectively. While FX significantly enhanced HAdV-5 transduction in both cell lines, transduction enhancement for AdT* was negligible compared to that of HAdV-5 (Fig. 1B and data not shown). Transduction assays were then performed with AdT* in the presence of immunocompetent C57BL/6 or immunocompromised Rag 2^−/−^ serum. As expected, C57BL/6 serum inhibited AdT* transduction both in the presence or absence of X-bp (Fig. 1C), confirming again the adenovirus neutralization properties of immunocompetent C57BL/6 serum in the absence of FX binding. In contrast, Rag 2^−/−^ serum enhanced AdT* transduction, and X-bp had no effect on this enhancement (Fig. 1C). These data therefore confirm that HAdV-5 predominantly uses a FX-independent pathway for transduction in the presence of immunocompromised Rag 2^−/−^ serum.

To investigate whether a FX-independent mechanism of HAdV-5 transduction is also observed in the presence of immunocompetent C57BL/6 serum, HAdV-5 transduction of A549 cells was assessed in the presence of C57BL/6 serum that had been preincubated at 56°C for 30 min to inactivate the complement system and thus inhibit adenovirus neutralization (17). Immunocompromised Rag 2^−/−^ serum was used as a nonneutralizing control serum. As expected, C57BL/6 and Rag 2^−/−^ serum not subjected to heat treatment significantly enhanced HAdV-5 transduction (2.2-fold and 3.3-fold, respectively), and addition of X-bp to C57BL/6 serum inhibited HAdV-5 transduction in contrast to Rag 2^−/−^ serum (Fig. 1D and E). Heat-treated C57BL/6 serum preincubated with X-bp failed to inhibit HAdV-5 transduction (Fig. 1D), similar to that observed with heat-treated Rag 2^−/−^ serum in the presence of X-bp (Fig. 1E), indicating that adenovirus neutralization was successfully inhibited by heat. Interestingly, exposure of C57BL/6 or Rag 2^−/−^ serum to heat inhibited the transduction-enhancing properties of both sera (Fig. 1D and E), suggesting a role for heat-labile factors in *in vitro* neutralization and transduction of adenoviral vectors. To confirm that FX is heat labile, HAdV-5 transduction of A549 cells was assessed in the presence of heat-treated hFX. While hFX enhanced HAdV-5 transduction by 2.9-fold, heat-treated hFX failed to enhance HAdV-5 transduction (Fig. 1F). These data suggest that both FX-dependent and -independent mechanisms of HAdV-5 transduction in the presence of mouse serum are mediated by heat-labile factors.

### Immunocompromised mouse serum enhances HAdV-5 transduction in cell lines expressing high levels of CAR in a FX-independent manner.

**FIG 2:**
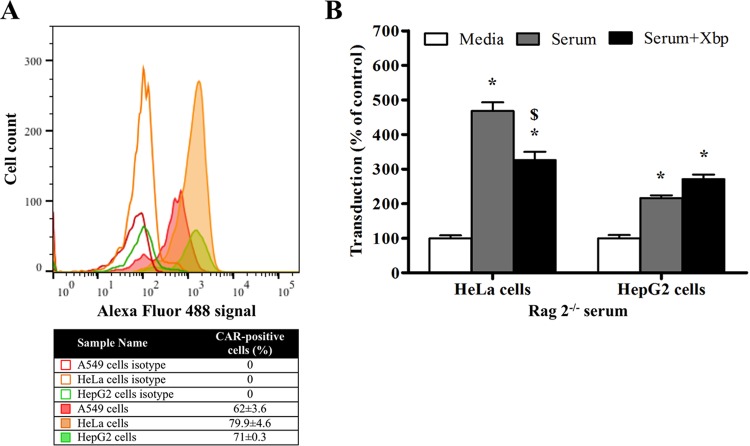
Adenoviral transduction in the presence of immunocompromised mouse serum in different cell lines expressing high levels of CAR. (A) CAR expression levels on cell plasma membrane were tested by flow cytometry in A549, HeLa, and HepG2 cells. CAR-positive cells are expressed as a percentage of the parental population and the mean of technical triplicates ± SEM. Representative data are shown. (B) HAdV-5 (2 × 10^10^ vp/ml) was incubated for 30 min at 37°C with serum-free (SF) media or 90% Rag 2^−/−^ serum in the presence or absence of X-bp (40 μg/ml). Adenovirus suspensions were added to HeLa or HepG2 cells (MOI, 1,000 vp/cell) and incubated at 37°C for 2 h. Then, the medium was replaced with media containing 2% FCS, and the cells were incubated for an additional 20 h. β-Galactosidase expression levels were quantified as relative light units (RLU) and normalized to the total milligrams of protein. The data represent pooled values from six (HeLa cells) or three (HepG2 cells) independent experiments with four replicates per condition. Values are shown as a percentage of the SF-medium-alone condition and expressed as the mean of the normalized values per experiment plus SEM. Repeated-measures ANOVA and *posthoc* Tukey's range test were applied. *, *P* < 0.05 versus matched controls; $, *P* < 0.05 versus serum.

We next investigated the role of CAR as a possible cellular receptor for the observed mouse serum-enhanced HAdV-5 transduction of cells *in vitro*. HAdV-5 transduction assays were performed in the presence of Rag 2^−/−^ serum and X-bp in HeLa and HepG2 cell lines, which express CAR at high levels on the plasma membrane, similar to A549 cells (Fig. 2A). For both HeLa and HepG2 cells, HAdV-5 transduction was enhanced by Rag 2^−/−^ serum, and addition of X-bp to Rag 2^−/−^ serum had little effect on transduction enhancement (Fig. 2B), equivalent to that observed earlier for A549 cells. These data indicate that a FX-independent pathway for HAdV-5 transduction is also present in other high-CAR-expressing cell lines in the presence of Rag 2^−/−^ serum, suggesting a possible role for CAR in the observed paradigm.

### Immunocompetent and immunocompromised mouse sera enhance HAdV-5 transduction *in vitro* in a CAR-dependent manner.

**FIG 3:**
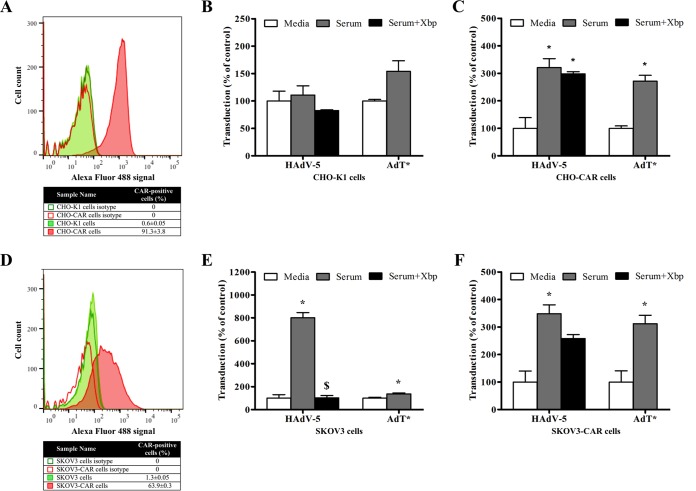
Adenoviral transduction in the presence of immunocompromised mouse serum in different cell lines engineered to express CAR. (A and D) CAR expression levels on cell plasma membrane were tested by flow cytometry in CHO-K1 and CHO-CAR cells (A) and SKOV3 and SKOV3-CAR cells (D). CAR-positive cells are expressed as a percentage of the parental population and the mean of technical triplicates ± SEM. Representative data are shown. (B, C, E, and F) HAdV-5 or AdT* (FX binding-deficient) (2 × 10^10^ vp/ml) was incubated for 30 min at 37°C with serum-free (SF) media or 90% Rag 2^−/−^ serum in the presence or absence of X-bp (40 μg/ml). Adenovirus suspensions were added to CHO-K1 (B), CHO-CAR (C), SKOV3 (E), or SKOV3-CAR (F) cells (MOI, 1,000 vp/cell) and incubated at 37°C for 2 h. Then, the medium was replaced with media containing 2% FCS, and the cells were incubated for an additional 20 h. β-Galactosidase expression levels were quantified as relative light units (RLU) and normalized to the total milligrams of protein. The data represent pooled values from three (B, C, and F) or four (E) independent experiments with four replicates per condition. Values are shown as a percentage of the SF-medium-alone condition and expressed as the mean of the normalized values per experiment plus SEM. Transduction values (RLU/mg of total protein) for HAdV-5 in the presence of SF media from a representative independent experiment are indicated below to add clarity on the magnitude of transduction levels: 4.8 × 10^4^ (CHO-K1 cells), 3.8 × 10^6^ (CHO-CAR cells). Repeated-measures ANOVA and *posthoc* Tukey's range test were applied. *, *P* < 0.05 versus matched controls; $, *P* < 0.05 versus serum.

To further assess the role of CAR in HAdV-5 transduction in the presence of immunocompromised mouse serum, transduction experiments were performed in CHO-K1 control cells and CHO cells stably expressing human CAR (hCAR) (CHO-CAR) (5) (Fig. 3A) in the presence of Rag 2^−/−^ serum and X-bp. Transduction of CAR-negative CHO-K1 cells with HAdV-5 was negligible (Fig. 3B), in agreement with previous reports (47), and Rag 2^−/−^ serum did not enhance transduction (Fig. 3B), suggesting that mouse FX (mFX) is unable to mediate transduction in CHO-K1 cells, as reported previously (48). Interestingly, CHO-CAR cells showed a transduction pattern (Fig. 3C) similar to that observed in A549 cells (Fig. 1A and C). The transduction enhancement for HAdV-5 in the presence of Rag 2^−/−^ serum was approximately threefold and unaffected by preincubation with X-bp (Fig. 3C), indicating that HAdV-5 transduction of CHO-CAR cells was not dependent on FX. In addition, AdT* transduction was efficiently enhanced by Rag 2^−/−^ serum in CHO-CAR cells (Fig. 3C). These data suggest that a FX-independent and CAR-mediated mechanism may be responsible for HAdV-5 transduction of CHO-CAR cells in the presence of Rag 2^−/−^ serum.

The role of CAR in Rag 2^−/−^ serum-enhanced HAdV-5 transduction was confirmed using SKOV3 cells (CAR^low^) and SKOV3 cells engineered to stably express hCAR (SKOV3-CAR) (Fig. 3D). Rag 2^−/−^ serum enhanced HAdV-5 transduction of SKOV3 cells by approximately eightfold (Fig. 3E). Incubation of Rag 2^−/−^ serum with X-bp completely inhibited the transduction-enhancing effects for HAdV-5 in SKOV3 cells (Fig. 3E), supporting a predominant role for FX in mediating SKOV3 cell transduction for HAdV-5 in agreement with a previous report (18). Furthermore, Rag 2^−/−^ serum enhanced AdT* transduction only minimally (Fig. 3E), again supporting the assertion that HAdV-5 transduction of SKOV3 cells in the presence of Rag 2^−/−^ serum is predominantly mediated by FX. Rag 2^−/−^ serum also enhanced HAdV-5 transduction of SKOV3-CAR cells (3.5-fold) (Fig. 3F). In contrast to SKOV3 cells, preincubation of Rag 2^−/−^ serum with X-bp only marginally decreased Rag 2^−/−^ serum-enhanced HAdV-5 transduction of SKOV3-CAR cells (Fig. 3F), indicating a minimal contribution of FX to serum-enhanced HAdV-5 transduction in this cell line. Despite the lack of FX binding, transduction of SKOV3-CAR cells with AdT* was enhanced threefold by Rag 2^−/−^ serum similar to that for HAdV-5 (Fig. 3F). These data support the evidence for a FX-independent but CAR-dependent mechanism of HAdV-5 transduction in the presence of immunocompromised mouse serum in CAR-expressing cells.

**FIG 4:**
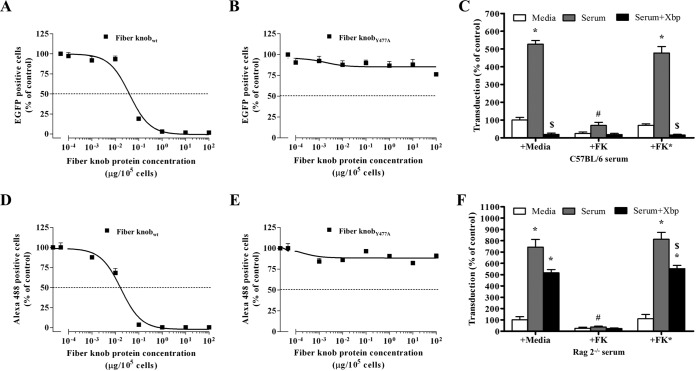
Role of CAR on adenoviral transduction in the presence of mouse serum. (A, B, D, and E) Characterization of soluble recombinant fiber knob (FK) binding to CAR. CHO-CAR cells were preincubated with FK or FK* (FK with a Y477A point mutation to impair binding to CAR) (0.0001 to 100 μg/10^5^ cells) at 4°C for 1 h to competitively inhibit HAdV-5-EGFP (A and B) or anti-CAR antibody (clone RmcB; Upstate, NY) (D and E) binding to CAR. FK-preincubated CHO-CAR cells were incubated with HAdV-5-EGFP (10 PFU/cell) for 1 h at 4°C, the medium was replaced with media containing 10% FCS, the cells were incubated for an additional 22 h at 37°C, and EGFP expression levels corresponding to transduced cells were quantified by flow cytometry (A and B). FK-preincubated CHO-CAR cells were incubated with anti-CAR antibody, and its binding was detected by flow cytometry with Alexa Fluor 488-labeled goat anti-mouse IgG antibody (D and E). (C and F) HAdV-5 (2 × 10^10^ vp/ml) was incubated for 30 min at 37°C with serum-free (SF) media, 90% C57BL/6 (C) or Rag 2^−/−^ (F) serum in the presence or absence of X-bp (40 μg/ml). Adenovirus suspensions were added to A549 cells (MOI, 1,000 vp/cell) that had been preincubated with SF media, soluble recombinant fiber knob (FK), or FK* at 1 μg/well. After 2 h of incubation at 37°C, the medium was replaced with 2% FCS-containing media, and the cells were incubated for an additional 20 h. β-Galactosidase expression levels were quantified as relative light units (RLU) and normalized to the total milligrams of protein. There were three technical replicates per condition in panels A, B, D, and E. The pooled data from three independent experiments with four replicates per condition are shown in panels C and F. Values are shown as a percentage of the SF-medium-alone condition and expressed as the mean of the normalized values per experiment plus SEM. Repeated-measures ANOVA and *posthoc* Tukey's range test were applied. *, *P* < 0.05 versus matched controls; $, *P* < 0.05 versus matched serum; #, *P* < 0.05 versus serum plus FK*.

To further interrogate HAdV-5 receptor usage, soluble recombinant HAdV-5 fiber knob (FK) or FK*, which contains the Y477A point mutation in the fiber DE loop to prevent binding to CAR (30, 49, 50), was used. Binding of FK or FK* to CAR on the plasma membrane was tested by performing competition assays for binding to CAR with HAdV-5-EGFP (EGFP stands for enhanced green fluorescent protein) or an anti-CAR antibody. While preincubation of CHO-CAR cells with FK inhibited HAdV-5-EGFP binding to cells at a median inhibitory concentration (IC_50_) of 0.04 μg/10^5^ cells (Fig. 4A), FK* did not reach IC_50_ even with excess levels of protein (100 μg/10^5^ cells) (Fig. 4B), confirming that FK* binding to CAR is substantially reduced. Similarly, incubation of CHO-CAR cells with an anti-CAR antibody following preincubation of cells with FK resulted in an IC_50_ of 0.017 μg/10^5^ cells (Fig. 4D). However, FK* failed to inhibit anti-CAR antibody binding even at concentrations up to 100 μg/10^5^ cells (Fig. 4E), confirming the reduced FK* binding to CAR. In order to block CAR, A549 cells were incubated with FK during HAdV-5 transduction in the presence of immunocompetent C57BL/6 serum (Fig. 4C) or immunocompromised Rag 2^−/−^ (Fig. 4F) serum. FK* was used as a negative control. C57BL/6 and Rag 2^−/−^ serum enhanced HAdV-5 transduction (Fig. 4C and F). Preincubation of C57BL/6 serum with X-bp inhibited HAdV-5 transduction (Fig. 4C), again supporting a role for FX binding HAdV-5 to protect it from neutralization. In contrast, X-bp only marginally decreased Rag 2^−/−^ serum-enhanced transduction (Fig. 4F), as previously observed (Fig. 1A). Importantly, preincubation of cells with wild-type FK but not FK* prevented both C57BL/6 and Rag 2^−/−^ serum-enhanced HAdV-5 transduction (Fig. 4C and F). These results indicate that blockade of CAR is sufficient to inhibit mouse serum-enhanced HAdV-5 transduction, supporting a role for CAR in transduction *in vitro* in the presence of mouse serum.

### A direct interaction with CAR is not necessary for HAdV-5 transduction in the presence of immunocompromised mouse serum.

**FIG 5:**
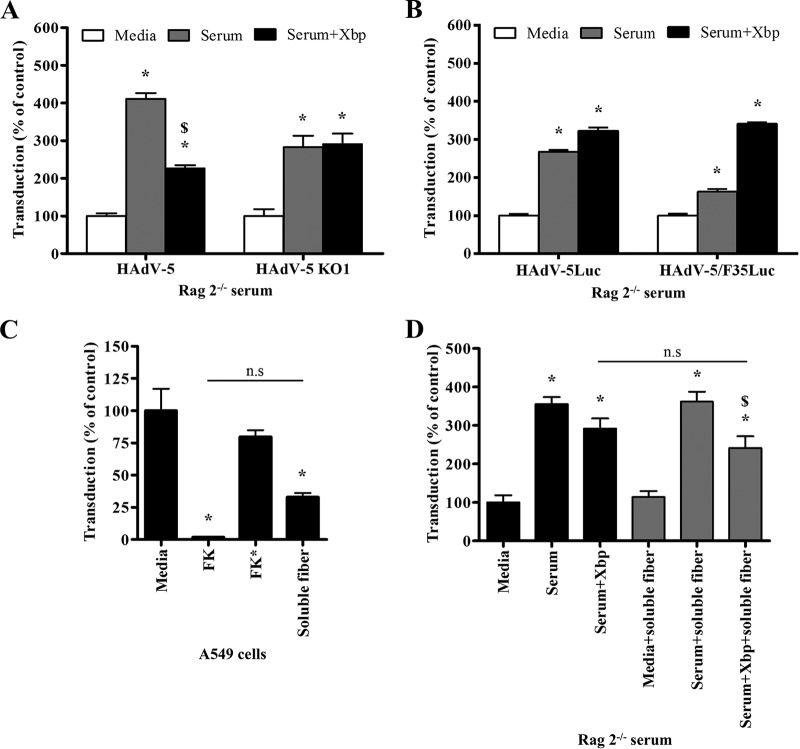
Role of HAdV-5 fiber in adenovirus transduction in the presence of immunocompromised mouse serum. (A, B, and D) HAdV-5, HAdV-5 KO1 (CAR binding greatly reduced), HAdV-5Luc, or HAdV-5/F35Luc (CAR binding-deficient) (2 × 10^10^ vp/ml) was incubated for 30 min at 37°C with serum-free (SF) media or 90% Rag 2^−/−^ serum in the presence or absence of X-bp (40 μg/ml). When indicated, Rag 2^−/−^ serum was preincubated with soluble fiber (63.13 μM) for 30 min at 37°C before the addition of HAdV-5 (D). Adenovirus suspensions were added to A549 cells (MOI, 1,000 vp/cell) and incubated at 37°C for 2 h. Then, the medium was replaced with media containing 2% FCS, and the cells were incubated for an additional 20 h. (C) A549 cells were incubated with HAdV-5 (MOI, 10,000 vp/cell) at 37°C for 3 h following preincubation of cells with serum-free (SF) media, soluble recombinant HAdV-5 fiber knob (FK), or FK* (Y477A point mutation to impair binding to CAR) at 0.2 μg/well or soluble HAdV-5 fiber at 0.59 μg/well. The medium was replaced with 10% FCS-containing media, and the cells were incubated for an additional 48 h. β-Galactosidase (A, C, and D) or luciferase (B) expression levels were quantified as relative light units (RLU) and normalized to the total milligrams of protein. Background chemiluminescence was subtracted from all values in panel C. There were four technical replicates per condition (C) and pooled data from four (A) or three (B and D) independent experiments with four replicates per condition. Values are shown as a percentage of the SF-medium-alone condition and expressed as the mean of the normalized values per experiment plus SEM (A, B, and D) or as the mean of values plus SEM (C). Repeated-measures ANOVA and *posthoc* Tukey's range test were applied. *, *P* < 0.05 versus matched controls; $, *P* < 0.05 versus serum; n.s, not significant.

Next, to assess whether HAdV-5 interaction with CAR in the presence of immunocompromised mouse serum is via a direct interaction through the fiber knob domain, transduction assays in A549 cells were performed in the presence of Rag 2^−/−^ serum using HAdV-5 KO1 (KO stands for knockout). HAdV-5 KO1 has selective greatly reduced binding to CAR via point mutations in specific residues within the fiber knob domain (S408E and P409A [29, 51]) to maintain structural integrity of the capsid and avoid impairment of other possible interactions that might take place with the HAdV-5 fiber. HAdV-5 KO1 transduction was enhanced 2.8-fold in the presence of Rag 2^−/−^ serum, both with and without X-bp (Fig. 5A). These results indicate that the enhanced transduction observed in the presence of mouse serum is FX independent and might not depend on direct interaction of HAdV-5 fiber knob with CAR.

To confirm that a direct interaction between HAdV-5 and CAR is not required in this setting and that the mechanism behind mouse serum-enhanced transduction does not involve a mouse serum protein(s) stabilizing the weak interaction of HAdV-5 KO1 fiber knob with CAR, a HAdV-5 vector containing the fiber of CAR binding-deficient HAdV-35 (HAdV-5/F35Luc [Luc stands for luciferase]) was used. HAdV-5/F35Luc transduction of A549 cells was enhanced in the presence of Rag 2^−/−^ serum with and without X-bp (Fig. 5B), indicating that transduction was FX independent and thus confirming that direct interaction between HAdV-5 and CAR is not required for Rag 2^−/−^ serum-enhanced transduction.

The observation that a direct interaction between HAdV-5 and CAR is not necessary for enhanced transduction in the presence of immunocompromised Rag 2^−/−^ serum (Fig. 5A and B), together with the observation that addition of soluble FK directly to cells did inhibit transduction (Fig. 4C and F), highlights the possibility that an additional factor present in the serum may bridge HAdV-5 to CAR in this setting. Moreover, the observation that HAdV-5/F35Luc transduction is enhanced by Rag 2^−/−^ serum in a FX-independent manner suggests that the HAdV-5 fiber might be dispensable for interactions with the mouse serum protein(s) required for HAdV-5 transduction in the presence of mouse serum. To further investigate this hypothesis, soluble HAdV-5 fiber was used. To confirm that soluble fiber binding sites were functional, the ability of soluble fiber to bind to CAR and block HAdV-5 transduction was confirmed (Fig. 5C). Next, transduction experiments with HAdV-5 on A549 cells were performed in the presence of Rag 2^−/−^ serum that had been preincubated with X-bp and/or soluble fiber. Rag 2^−/−^ serum preincubated with soluble fiber significantly enhanced HAdV-5 transduction of A549 cells by 3.6-fold, and X-bp only marginally decreased the enhanced transduction, similar to that observed with untreated control Rag 2^−/−^ serum (Fig. 5D). Importantly, preincubation of Rag 2^−/−^ serum with soluble fiber failed to inhibit serum-enhanced HAdV-5 transduction in the presence of X-bp (Fig. 5D), providing further evidence that the HAdV-5 fiber might be dispensable for interactions with the mouse serum protein(s) involved in HAdV-5 transduction in the presence of mouse serum.

### FVII, FIX, and PC are not required for HAdV-5 transduction in the presence of mouse plasma.

**FIG 6:**
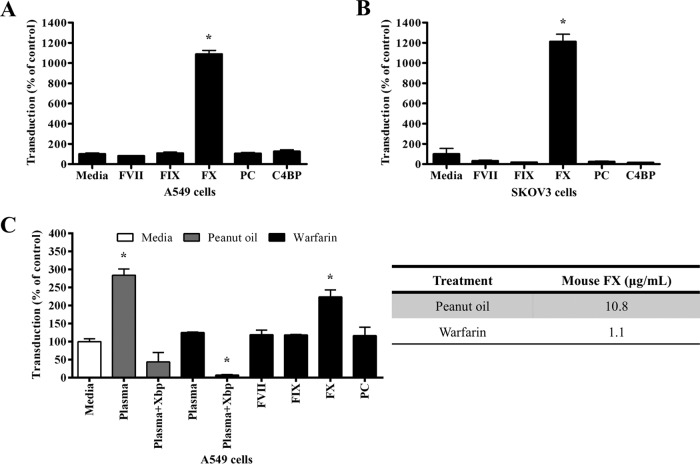
Effect of FVII, FIX, FX, PC, and C4BP on HAdV-5 transduction *in vitro*. (A and B) HAdV-5 (2 × 10^10^ vp/ml) was incubated for 30 min at 37°C with serum-free (SF) media containing human FVII (0.5 μg/ml), FIX (5 μg/ml), FX (10 μg/ml), PC (4 μg/ml), or C4BP (200 μg/ml). Adenovirus suspensions were added to A549 (A) or SKOV3 (B) cells (MOI, 10,000 vp/cell) and incubated at 37°C for 2 h. (C) HAdV-5 (2 × 10^10^ vp/ml) was incubated for 30 min at 37°C with SF media or 90% plasma of peanut oil- or warfarin-treated C57BL/6 mice in the presence or absence of X-bp (40 μg/ml) or plasma supplemented with human FVII (0.5 μg/ml), FIX (5 μg/ml), FX (10 μg/ml), or PC (4 μg/ml). Adenovirus suspensions were added to A549 cells (MOI, 1,000 vp/cell) and incubated at 37°C for 2 h. The medium was then replaced with media containing 2% FCS, and the cells were incubated for an additional 20 h (A to C). β-Galactosidase expression levels were quantified as relative light units (RLU) and normalized to the total milligrams of protein. The relative concentration of FX in plasma from peanut oil- or warfarin-treated C57BL/6 mice was quantified by an ELISA (C). The data represent pooled values from three independent experiments with four replicates per condition. Values are shown as a percentage of the SF-medium-alone condition and expressed as the mean of the normalized values per experiment plus SEM. Repeated-measures ANOVA and *posthoc* Tukey's range test were applied. *, *P* < 0.05 versus SF-medium-alone condition.

To investigate which circulating mouse blood protein(s) might be involved in enhancing HAdV-5 transduction *in vitro* in the presence of mouse serum, the ability of FVII, FIX, FX, PC, or C4BP to enhance HAdV-5 transduction of A549 cells was assessed. CAR^low^ SKOV3 cells were used as a negative control. As expected, FX enhanced HAdV-5 transduction of A549 cells (Fig. 6A) and SKOV3 cells (Fig. 6B). In contrast, FVII, FIX, PC, and C4BP failed to enhance HAdV-5 transduction (Fig. 6A and B).

To further assess whether the circulating mouse blood protein(s) involved in mouse serum-enhanced HAdV-5 transduction requires the presence of other factors present in mouse serum to enhance adenovirus transduction, HAdV-5 transduction of A549 cells was assessed in the presence of plasma from C57BL/6 mice pretreated with warfarin to deplete vitamin K-dependent coagulation factors (9) and supplemented with FVII, FIX, FX, or PC. Plasma from peanut oil-treated C57BL/6 was used as a control. Warfarin treatment reduced plasma FX levels from physiological levels of 10.8 to 1.1 μg/ml (Fig. 6C). As expected, plasma from control C57BL/6 mice significantly enhanced HAdV-5 transduction of A549 cells (2.8-fold) and addition of X-bp substantially reduced HAdV-5 transduction compared to the control (Fig. 6C). Plasma from warfarin-treated C57BL/6 mice failed to enhance HAdV-5 transduction (Fig. 6C), which suggests that a vitamin K-dependent coagulation factor(s) might be responsible for the observed mouse plasma-enhanced HAdV-5 transduction. Addition of X-bp to warfarin-treated C57BL/6 plasma substantially inhibited HAdV-5 transduction (Fig. 6C). Of the tested coagulation factors, only addition of FX to warfarin-treated C57BL/6 plasma rescued mouse plasma-enhanced HAdV-5 transduction (Fig. 6C), indicating that FX is required for mouse plasma-enhanced transduction in contrast to FVII, FIX, and PC.

## DISCUSSION

In this study, the interactions that take place between HAdV-5 and host cells *in vitro* in the presence of immunocompetent or immunocompromised mouse serum were investigated. Blockade of HAdV-5–FX interactions with X-bp or the use of a FX binding-deficient HAdV-5 (AdT*) demonstrated that when HAdV-5 is exposed to immunocompetent mouse serum, the use of FX as a protective “shield” from “immune attack” is essential to allow adenoviral transduction, in agreement with previous findings (17–19). However, the contribution of FX to HAdV-5 transduction *in vitro* in the presence of immunocompromised mouse serum lacking the mediators for “immune attack” is minimal. Instead, HAdV-5 seems to predominantly follow a FX-independent mechanism to transduce cells. The use of two different strains of immunocompromised mice (Rag 2^−/−^ and NSG) showed that the FX-independent pathway of adenoviral transduction after exposure to mouse serum might be conserved across mouse strains. Furthermore, blockade of CAR with soluble recombinant HAdV-5 fiber knob and the use of CHO-CAR cells and SKOV3 cells expressing hCAR showed that the alternative FX-independent mechanism that mediates HAdV-5 transduction *in vitro* in the presence of mouse serum is dependent on CAR. Transduction experiments performed with HAdV-5 KO1 or CAR binding-deficient HAdV-5/F35Luc in the presence of immunocompromised Rag 2^−/−^ serum showed that direct interaction of HAdV-5 with CAR is not required for HAdV-5 transduction in the presence of mouse serum. This suggests the existence of a factor(s) present in Rag 2^−/−^ serum that might bridge HAdV-5 to CAR for enhancement of cell transduction. Moreover, the use of HAdV-5/F35Luc together with the assessment of HAdV-5 transduction following incubation of Rag 2^−/−^ serum with soluble HAdV-5 fiber showed that the HAdV-5 fiber might be dispensable for the use of the novel CAR-mediated pathway. Supplementation of vitamin K-dependent coagulation factor-depleted mouse plasma with individual coagulation factors showed that FVII, FIX, and PC are not required for HAdV-5 cell transduction via the novel CAR-mediated pathway. Instead, assessment of HAdV-5 transduction in the presence of mouse serum that had been heat treated suggested a role for a heat-labile factor(s) in this setting. Further experiments using inhibitors for specific components of the complement system or depleting serum of IgM antibodies would provide valuable information in defining the mechanism mediating HAdV-5 transduction in the presence of immunocompetent C57BL/6 serum.

In gene therapy, it is essential that the gene transfer vector reaches the target organ while avoiding the activation of immune responses and toxic side effects. Thus, when administering adenoviral vectors intravenously, knowledge about adenoviral behavior and its interactions with blood proteins is crucial. Adenoviruses are pathogens that can cause a wide range of infections, such as conjunctivitis, tonsillitis, respiratory tract or ear infections, and gastroenteritis. To fight infections, the host organism has developed a series of physical and chemical barriers as well as the immune system; a network of molecules, cells, and organs working together to protect the organism against threats. Despite the effectiveness of the immune system against pathogens, adenoviruses have evolved to take advantage of some of the interactions that take place in the host organism. An example is coagulation factor X, which binds to HAdV-5 in the bloodstream and is thought to be used as a protective “shield” against the attack on the capsid by the immune system (17–19). Simultaneously, HAdV-5 also exploits this interaction with FX to engage with HSPG on the surface of cells allowing viral entry (9, 11, 13, 14). While HAdV-5 can use HSPGs as cell receptors for transduction *in vitro* in the presence of FX (13, 20) and *in vivo* when administered through the vasculature (9), other studies showed that HAdV-5 liver transduction in immunocompromised mice lacking IgM antibodies or complement was not impaired by ablation of HAdV-5–FX interaction (17). Also, recent studies using mice lacking HS report that HSPGs are dispensable for HAdV-5 liver transduction (20). These studies suggest that adenovirus might follow other cell entry pathways when the FX-mediated pathway is not available.

Evidence that CAR is mainly expressed on the basolateral side of the plasma membrane (52, 53), an area not accessible for nonreplicating adenoviruses, has historically brought into question the biological relevance of HAdV-5–CAR interactions during cell entry. CAR is part of the immunoglobulin superfamily encoded by a highly conserved gene that via alternative splicing can generate five alternative transcripts corresponding to three soluble isoforms (54, 55) and two transmembrane isoforms (CAR^Ex7^ and CAR^Ex8^) that differ in their carboxy terminus (56). CAR^Ex7^, the most abundant isoform, localizes to the basolateral surface of polarized epithelia and is involved in cell-cell adhesion (57). Interestingly, a recent study showed that the CAR^Ex8^ isoform localizes to the apical membrane (56). Importantly, the expression of apical CAR is stimulated by the proinflammatory cytokine and neutrophil chemoattractant interleukin 8 (IL-8) and was associated with promotion of adenovirus cell entry (58). These reports together with the evidence for a CAR-dependent mechanism of HAdV-5 transduction of cells *in vitro* following exposure of virions to mouse serum might begin to unravel alternative transduction mechanisms defining HAdV-5 liver tropism following intravascular delivery.

Here, the use of SKOV3 cells expressing low levels of CAR confirmed that when access to CAR is limited, HAdV-5 may transduce cells via the FX-mediated pathway in the presence of Rag 2^−/−^ serum. These results suggest that the use of either the FX-dependent pathway or the novel CAR-mediated pathway for HAdV-5 transduction in the presence of Rag 2^−/−^ serum may depend on the availability of the corresponding receptors (CAR and HSPG). This observation is in agreement with previous studies showing that other adenovirus serotypes such as HAdV-35 can use FX, although with lower affinity than that of HAdV-5 and that FX might compete with CD46 for binding to HAdV-35 (59). Our data suggest that the FX-dependent pathway and the novel CAR-mediated pathway for HAdV-5 transduction *in vitro* in the presence of immunocompromised Rag 2^−/−^ serum may be available simultaneously. Based on these data, however, the CAR-mediated pathway is the predominant route HAdV-5 follows for transduction of cells *in vitro* in this setting. It still remains unclear what the determining factors are for the use of one pathway or the other pathway or if they influence each other. For instance, the relative abundance of HSPGs or CAR on the plasma membrane might affect which pathway is used (48). Also, the differential levels of expression of HSPGs and CAR in different cell lines might have an impact on receptor usage and account for the differences observed on HAdV-5 transduction between cell lines or tissues. The affinity of HAdV-5 for individual components of the transduction pathways identified (e.g., receptors, bridging molecules, etc.) might also determine the route of entry that HAdV-5 follows during cell transduction. For example, a previous report indicated that differences in the affinity of HAdV-5–hFX or HAdV-5–mFX complexes for HSPGs affected their ability to enhance HAdV-5 transduction in cultured cells (48). Nevertheless, despite the CAR amino acid sequence being highly conserved (6, 60), mouse and human CAR might differ in their interactions with mouse serum proteins. Thus, the use of mouse cells expressing mouse CAR in HAdV-5 transduction studies in the presence of mouse serum would provide useful mechanistic data on the use of the novel CAR-mediated pathway for HAdV-5 transduction.

Experiments using other translationally relevant animal models or human samples are fundamental in investigating the mechanisms mediating adenovirus transduction and their implications for the clinic. Interestingly, a recent study showed high variability in the effect of human serum (with no preexisting neutralizing human IgG [hIgG] antibodies) on HAdV-5 *in vitro* neutralization and transduction (19), which is in contrast to data from mouse models, highlighting the limitations of studies in small animal models. This study demonstrated a protective role for FX binding to HAdV-5 capsids against adenovirus neutralization in 56% of human serum samples analyzed and of the remaining 44% that did not neutralize HAdV-5 in the absence of FX binding, some individual serum samples enhanced transduction in a completely FX-dependent manner and others enhanced transduction in a partially FX-dependent manner (19). Further studies assessing the effect of IgM-depleted human serum with no preexisting neutralizing hIgG antibodies on FX binding-deficient HAdV-5 vectors will provide valuable information to dissect the underlying mechanisms of adenovirus tropism in humans following systemic delivery of vectors.

**FIG 7:**
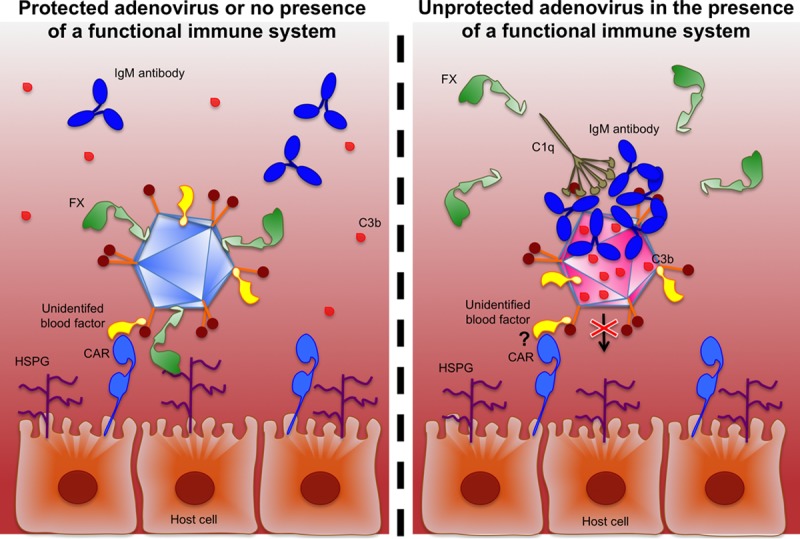
Schematic representation of the suggested model of HAdV-5 cell transduction in the presence of mouse serum. HAdV-5 may use FX for protection against adenovirus neutralization in the presence of immunocompetent mouse serum. In the absence of adenovirus neutralization and in the presence of mouse serum, HAdV-5 may transduce host cells through a coagulation factor X (FX)-dependent mechanism and use an unidentified blood protein for transduction of host cells via the coxsackievirus and adenovirus receptor (CAR). HSPG, heparan sulfate proteoglycans; C1q, complement component 1q; C3b, complement component 3b.

Taken together, the data suggest that HAdV-5 can make use of different host cell receptors (such as HSPGs and CAR) by binding to bridging molecules present in mouse serum (Fig. 7). Receptor availability and affinity in individual cells may determine the entry pathway followed. Our findings have implications for understanding HAdV-5–host interactions and for the development of safer and more efficient adenoviral gene transfer vectors.

## MATERIALS AND METHODS

### Ethics statement.

All animal experiments were approved by the University of Glasgow Animal Procedures and Ethics Committee and performed under UK Home Office license (PPL 60/4429) in strict accordance with UK Home Office guidelines. Animals were housed under controlled environmental conditions at ambient temperature with 12-h light/dark cycles. Mice were fed standard chow, and water was provided *ad libitum*.

### Immortalized cell lines.

Adherent HEK293 cells (human embryonic kidney; ATCC CRL-1573), HeLa cells (human cervix adenocarcinoma; ATCC CCL-2), and HepG2 cells (human liver hepatocellular carcinoma; ATCC HB-8065) were cultured in minimum essential medium (MEM) (GIBCO by Life Technologies) supplemented with 100 U/ml penicillin and 100 μg/ml streptomycin (GIBCO by Life Technologies), 10% (vol/vol) fetal calf serum (FCS) (PAA Laboratories), 1 mM sodium pyruvate (Sigma-Aldrich, UK) and 2 mM l-glutamine (Invitrogen by Life Technologies) at 37°C and 5% CO_2_. Adherent A549 cells (human lung carcinoma; ATCC CCL-185), SKOV3 cells (human ovary adenocarcinoma; ATCC HTB-77), and SKOV3-CAR cells (SKOV3 cells engineered to stably overexpress red fluorescent protein [RFP]-tagged human coxsackievirus and adenovirus receptor [hCAR]) were grown in Roswell Park Memorial Institute medium (RPMI 1640) (GIBCO by Life Technologies) supplemented with 100 U/ml penicillin, 100 μg/ml streptomycin, 10% (vol/vol) FCS, 1 mM sodium pyruvate, and 2 mM l-glutamine at 37°C 5% CO_2_. SKOV3-CAR cell medium was also supplemented with Geneticin (Gibco) at 1,000 ng/μl. Adherent CHO-K1 cells (Chinese hamster ovary; ATCC CCL-61) and CHO-CAR cells (CHO cells transfected to stably express human CAR [hCAR] [5] [a kind gift from George Santis, King's College London School of Medicine, London, UK]) were grown in Ham's F-10 nutrient mixture medium (GIBCO by Life Technologies) containing 1 mM l-glutamine and 1 mM sodium pyruvate supplemented with 100 U/ml penicillin, 100 μg/ml streptomycin, and 10% (vol/vol) FCS at 37°C 5% CO_2_. The stable cell line SKOV3-CAR was generated by transfecting a plasmid containing a RFP-tagged hCAR construct (61) (a gift from George Santis, King's College London School of Medicine, London, UK) into SKOV3 cells as described previously (62), and cell clones were selected with Geneticin at 1,000 ng/μl.

### Adenoviral vector construction.

Genetic characteristics of all adenoviral vectors used are described in Table 1. HAdV-5-EGFP has been previously described (63). HAdV-5HVR5*HVR7*E451Q (termed AdT*) (12) has seven point mutations in hexon HVRs 5 and 7 to ablate binding to coagulation FX (T270P and E271G in hexon HVR 5 and I421G, T423N, E424S, L426Y, and E451Q in HVR 7). HAdV-5/F35Luc is a HAdV-5 vector containing the fiber of HAdV-35 (59), and HAdV-5Luc (59) and HAdV-5/F35Luc encode the firefly luciferase reporter gene under the cytomegalovirus immediate early promoter (CMV-IEP). HAdV-5 KO1 was designed and generated based on the AdEasy E1/E3-deleted HAdV-5 adenoviral vector system (Stratagene by Agilent Technologies). As with HAdV-5 and AdT*, HAdV-5 KO1 carries the Escherichia coli lacZ reporter gene, which encodes the bacterial cytoplasmic β-galactosidase, under the CMV-IEP in place of the viral *E1* early gene. HAdV-5 KO1 has two point mutations (S408E and P409A [49, 51]) in the AB loop region of the fiber knob domain that greatly reduce binding to CAR. The fiber open reading frame (ORF) containing the KO1 mutation was excised from pAdT*KO1, a plasmid containing the AdT* vector genome (12) with a mutated fiber ORF sequence. Briefly, pAdT*KO1 was generated by homologous recombination with a shuttle vector engineered to contain the S408E and P409A mutations. The fiber ORF from pAdT*KO1 was excised and inserted between the SpeI and MfeI sites 5′ and 3′ to the fiber ORF of pShuttle-KO1-AAA (Agilent Technologies), respectively, to create pShuttle-KO1. pAdEasy1 (Agilent Technologies) and pShuttle-CMV-*LacZ* (Agilent Technologies) that had been linearized with PmeI, were subjected to homologous DNA recombination to generate pAdEasy1-CMV-*LacZ*. Then, pShuttle-KO1 was used to introduce the fiber ORF containing the KO1 mutation (excised with AscI and PacI sites 5′ and 3′ of the fiber ORF, respectively) into the pAdEasy1-CMV-*LacZ* (linearized with SpeI) by homologous DNA recombination to generate pAdEasy1-CMV-*LacZ*-KO1 (HAdV-5 KO1 genome).

**TABLE 1:** Description of adenoviral vectors^*a*^

Adenoviral vector	Receptor recognition characteristic	Protein targeted for mutation: region and domain mutated	Genetic mutation(s)
HAdV-5	Native tropism	None	None
HAdV-5-EGFP	Native tropism	None	None
HAdV-5Luc	Native tropism	None	None
AdT* (HAdV-5HVR5*HVR7*E451Q)	FX-binding deficiency	Hexon: HVR 5 and 7	T270P and E271G on HVR 5 and I421G, T423N, E424S, L426Y, and E451Q on HVR 7
HAdV-5 KO1	Greatly reduced CAR binding	Fiber: AB loop region of the fiber knob domain	S408E and P409A
HAdV-5/F35Luc	CAR-binding deficiency	Fiber	Serotyping of HAdV-5 with HAdV-35 fiber

a HAdV-5, human adenoviral vector serotype 5; HVR, hypervariable region; FX, coagulation factor X; CAR, coxsackievirus and adenovirus receptor.

### Adenoviral vector production.

HAdV-5, AdT*, HAdV-5 KO1, HAdV-5Luc, and HAdV-5/F35Luc vectors were propagated and purified as described previously (18, 62). HAdV-5-EGFP has been previously described (63). Quality control of all adenoviral vectors was performed by sequencing of the hexon HVR or fiber knob regions encoding the mutations (primers for hexon HVR 5/HVR 7 [5′-CTCAGTGGTACGAAACTGAA-3′], hexon HVR 7 [5′-CTATGTGGAATCAGGCTGTT-3′], and fiber knob AB loop KO1 mutation [5′-AATGCACCAAACACAAATCC-3′]) after amplification of specific sequences by PCR (primers for hexon-F [F stands for forward] [5′-CCCGCTTTCCAAGATGGCTA-3′], hexon-R [R stands for reverse] [5′-GTTGGCGGGTATAGGGTAGA-3′], fiber-F [5′-ACTGCCACTGGTAGCTTGGG-3′], and fiber-R [5′-TGGCCAGCTGGTTTAGGATG-3′]) using the BigDye Terminator v3.1 cycle sequencing kit (Applied Biosystems by Life Technologies). Capillary electrophoresis was performed in a 3730 DNA analyzer (Applied Biosystems by Thermo Fisher Scientific) using 3730 data collection v3.0 software. Titer determination of viral particles (vp)/ml was performed with the microbicinchoninic acid (BCA) protein assay (Pierce, Thermo Scientific, USA) using the conversion factor 1 μg protein = 4 × 10^9^ vp. The number of PFU/ml was calculated by endpoint dilution assay (62). Adenoviral capsid composition and integrity of capsid structure were confirmed by silver staining (using a Thermo Scientific kit according to the manufacturer's instructions). The laser-based nanoparticle tracking analysis (NTA) by NanoSight was used to characterize the size of adenoviral particles from pure preparations with NanoSight NTA v2.3 software.

### Construction of soluble fiber knob expression vectors and recombinant protein purification.

The soluble fiber knob_wt_ (wt stands for wild type) expression vector construction has been described before (63). The point mutation Y447A in the fiber DE loop to impair binding to CAR (30, 49, 50) was introduced into pQE30-Knob_wt_ vector by mutagenic PCR using primers MutY477A-F (Mut stands for mutant) (5′-TTCCTGGACCCAGAAGCTTGGAACTTTAGAAAT-3′) and MutY477A-R (5′-ATTTCTAAAGTTCCAAGCTTCTGGGTCCAGGAA-3′) as described previously (64). Sequences were verified by sequencing using primers pQE-F (5′-CGGATAACAATTTCACACAG-3′) and pQE-R (5′-GTTCTGAGGTCATTACTGG-3′). Positive clones were transformed into SG13009 (pREP4) chemically competent E. coli (Qiagen) for protein expression as described previously (63), and His-tagged soluble recombinant fiber knob_wt_ (FK) and fiber knob_Y477A_ (FK*) were purified by affinity chromatography as before (63).

### Characterization of soluble fiber knob binding to CAR.

In order to confirm the presence or absence of binding of FK or FK* to CAR, respectively, these proteins were used to compete with HAdV-5-EGFP or primary mouse monoclonal anti-CAR antibody (clone RmcB; Upstate, NY) for binding to CAR on the plasma membrane of CHO-CAR cells. Increasing concentrations of FK or FK* (0.0001 μg/10^5^ cells to 100 μg/10^5^ cells) were preincubated with 1 × 10^5^ CHO-CAR cells for 1 h at 4°C as described previously (64). Next, cells were incubated with HAdV-5-EGFP (10 PFU/cell) for 1 h at 4°C and washed with phosphate-buffered saline (PBS), the medium was replaced with medium containing 10% FCS, and cells were incubated for further 22 h at 37°C and 5% CO_2_. In a separate experiment, cells pretreated with FK or FK* (0.0001 to 100 μg/10^5^ cells) were incubated with primary mouse monoclonal anti-CAR antibody (10 μg/ml), and binding was detected using Alexa Fluor 488-labeled goat anti-mouse IgG antibody (Life Technologies by Thermo Fisher Scientific) at 16 μg/ml as described in the section below. EGFP expression levels corresponding to transduced cells or Alexa Fluor 488-positive cells were detected by flow cytometry as described previously (64). Inhibition is expressed as the percentage of EGFP-positive or Alexa Fluor 488-positive cells treated with fiber knob relative to the fluorescence detected from a fiber knob-untreated control. Median inhibitory concentration (IC_50_) values were calculated by nonlinear regression analysis on a dose-response curve using GraphPad Prism v3.03 (GraphPad Software, San Diego, CA, USA).

### Assessment of CAR expression levels by flow cytometry.

Expression levels of CAR on the cell membrane were measured by flow cytometry. Cultured cells were washed with Dulbecco's calcium- and magnesium-free PBS (DPBS), dissociated with citric saline and resuspended in ice-cold serum-free (SF) media to a concentration of 4 × 10^6^ cells/ml. A total of 2 × 10^5^ cells were incubated with an equal volume of primary mouse monoclonal anti-CAR antibody (clone RmcB; Upstate, NY) or mouse IgG isotype control (both at 5 μg/ml in ice-cold SF media) for 30 min at 4°C in triplicate. The cells were washed twice with SF media, incubated with secondary antibody (Alexa Fluor 488-labeled goat anti-mouse IgG; Life Technologies by Thermo Fisher Scientific) for 30 min at 4°C (4 μg/ml in ice-cold SF media), washed twice with SF media, and resuspended in 150 μl of ice-cold SF media for analysis. A BD FACSCanto II flow cytometer and BD FACSDiva v6.1.3 software were used for analysis and FlowJo single cell analysis v10.1 software for graphical representation. Viable cells were gated on the basis of forward and side light scatter profiles, with a minimum of 10,000 gated events analyzed per sample.

### Adenoviral transduction assay in the presence of blood factors, soluble recombinant HAdV-5 FK, or soluble HAdV-5 fiber.

A549 or SKOV3 cells were seeded at 1 × 10^4^ cells/well on 96-well culture plates and incubated overnight at 37°C and 5% CO_2_. Adenoviral vectors (2 × 10^10^ vp/ml) were incubated for 30 min at 37°C with 50 μl SF media containing human FVII (0.5 μg/ml), FIX (5 μg/ml), FX (10 μg/ml), PC (4 μg/ml), or C4BP (200 μg/ml) (Cambridge Bioscience, UK). The cells were washed with SF media, adenovirus suspensions were diluted 20-fold in SF media, and 100-μl portions of the adenovirus suspensions were added to cells (multiplicity of infection [MOI] of 10,000 vp/cell) and incubated at 37°C for 2 h. Then, medium was replaced with media containing 2% FCS, and the cells were incubated for further 20 h, washed with DPBS, and lysed with 100 μl reporter lysis buffer (Promega, USA). When indicated, human coagulation FX (hFX) was preincubated at 56°C for 30 min in 50 μl SF media prior to addition of adenoviral vectors, adenovirus suspensions were diluted 200-fold in SF media, and cells were incubated with an MOI of 1,000 vp/cell. For transduction assays to assess adenovirus binding to FX, cultured cells were transduced with an MOI of 1,000 vp/cell of adenoviral vectors for 3 h at 37°C and 5% CO_2_ in the presence or absence of hFX at a working concentration of 10 μg/ml. After the 3 h of incubation, medium was replaced with 10% FCS-containing media, and cells were incubated for an additional 48 h prior to cell lysis. For HAdV-5 fiber knob or fiber competition assays, cells were incubated for 30 min at 4°C with FK (0.2 μg/well), FK* (0.2 μg/well), or soluble HAdV-5 fiber (0.59 μg/well) (kindly provided by John H. McVey, University of Surrey, Guildford, Surrey, UK) in 50 μl SF media prior to addition of 10,000 vp/cell adenoviral vectors in an equal volume of SF media and incubation for 3 h at 37°C. After the 3 h of incubation, medium was replaced with 10% FCS-containing media, and cells were incubated for an additional 48 h prior to cell lysis. β-Galactosidase expression levels were quantified by Galacto-Light Plus β-galactosidase reporter gene assay (Life Technologies by Thermo Fisher Scientific, UK). Values were expressed as relative light units (RLU) and normalized to the total milligrams of protein, which were determined using the BCA protein assay (Pierce, Thermo Scientific, USA).

### Adenoviral transduction assay in the presence of mouse serum.

Cells were seeded at 1 × 10^4^ cells/well on 96-well culture plates and incubated overnight at 37°C and 5% CO_2_. Fresh serum from C57BL/6 (Harlan Laboratories, UK), Rag 2^−/−^ [B6(Cg)-*Rag2*^*tm1.1Cgn*^/J], or NSG (NOD-scid-gamma [NOD.Cg-*Prkdc*^scid^ *Il2rg*^tm1Wjl^/SzJ]) mice was separated from whole blood by centrifugation at 5,000 × *g* for 10 min at 4°C after the formation of a clot in a BD capillary blood collection microtainer tube (Fisher Scientific, UK) and stored at 4°C. When plasma was required, a BD capillary blood collection microtainer tube with lithium heparin designed for plasma collection was used (Fisher Scientific, UK). Adenoviral vectors (2 × 10^10^ vp/ml) were incubated for 30 min at 37°C with 50 μl SF media, fresh mouse serum, or serum preincubated with X-bp (40 μg/ml) at room temperature for 10 min. X-bp is a molecule that binds to the FX GLA domain, inhibiting its binding to HAdV-5 (42). Cells were washed with SF media, adenoviral suspensions were diluted 200-fold in SF media, and 100-μl portions of the adenoviral suspensions were added to the cells (MOI of 1,000 vp/cell). The cells were incubated at 37°C and 5% CO_2_ for 2 h, medium was replaced with 2% FCS-containing media, and cells were incubated for an additional 20 h prior to cell lysis. β-Galactosidase activity was quantified as described above, luciferase activity was measured using the luciferase assay (Promega, UK), and values were normalized to the total milligrams of protein as described above. When indicated, mouse serum was preincubated with soluble HAdV-5 fiber (63.13 μM) for 30 min at 37°C following addition of X-bp and prior to addition of adenoviral vectors or preincubated at 56°C for 30 min to inhibit the complement system prior to addition of X-bp as described previously (17). When mouse plasma was used, the relative concentration of FX in plasma from mice treated with peanut oil or warfarin (9, 65) was quantified by an enzyme-linked immunosorbent assay (ELISA) as previously described (18), and 90% plasma of peanut oil- or warfarin-treated mice was supplemented with human FVII (0.5 μg/ml), FIX (5 μg/ml), FX (10 μg/ml), or PC (4 μg/ml) prior to addition of X-bp. For soluble recombinant HAdV-5 fiber knob assays, cells were incubated for 30 min at 4°C with 1 μg/well of FK or FK* in 50 μl of SF media prior to adenovirus addition, and 50-μl portions of adenoviral suspensions (diluted 100-fold in SF media) were added to cells (MOI of 1,000 vp/cell).

### Statistical analysis.

Unpaired Student's *t* test or repeated-measures analysis of variance (ANOVA) and Tukey's range test for *posthoc* pairwise comparisons of groups were performed for significance assessment using GraphPad Prism v5.0 (GraphPad Software, San Diego, CA, USA), as specified for each analysis. A *P* value of <0.05 was considered statistically significant. *In vitro* data are shown as a percentage of one of the conditions (specified for each experiment) and expressed as the mean of values or of the normalized values per experiment ± standard error of the mean (SEM). Three or more independent experiments were performed in quadruplicate for each condition unless otherwise stated.
